# Prevalence of Human Papillomavirus Infection and Cervical Abnormalities among Women Attending a Tertiary Care Center in Saudi Arabia over 2 Years

**DOI:** 10.3390/tropicalmed8120511

**Published:** 2023-11-30

**Authors:** Layla Faqih, Lama Alzamil, Esraa Aldawood, Sarah Alharbi, Moammer Muzzaffar, Amani Moqnas, Heba Almajed, Ahmed Alghamdi, Mohammed Alotaibi, Sultan Alhammadi, Yazeed Alwelaie

**Affiliations:** 1 Department of Clinical Laboratories Sciences, College of Applied Medical Sciences, King Saud University, Riyadh 12372, Saudi Arabia; 2 Pathology and Clinical Laboratory Medicine Administration, King Fahad Medical City, Riyadh 11525, Saudi Arabia; 3 Infection, Immunity & Respiratory Medicine, School of Biological Sciences, University of Manchester, Manchester M13 9PL, UK; 4 Translation Health Sciences, Bristol Medical School, University of Bristol, Bristol BS1 3NY, UK

**Keywords:** HPV infection, cervical cancer, HPV epidemiology

## Abstract

Human papillomavirus (HPV) genotype distribution varies according to the assessment method and the population targeted. This study aimed to assess HPV infection prevalence in women aged 23 to 82 with abnormal cytology attending King Fahad Medical City (KFMC), Riyadh, Saudi Arabia, using retrospective data collected from January 2021 to December 2022. Cytological distribution included 155 samples of atypical squamous cells of undetermined significance (ASCUS) (n = 83), low-grade squamous intraepithelial lesion (LSIL) (n = 46), high-grade squamous intraepithelial lesion (HSIL) (n = 14), atypical squamous cells cannot exclude high-grade squamous intraepithelial lesion (ASC-H) (n = 10), and squamous cell carcinoma (SCC) (n = 2). All samples were submitted to HPV detection and genotyping using Xpert HPV assay specimens. The most prevalent epithelial abnormalities were ASCUS (53.50%). Positive HPV infection results were observed in 52.9% of the samples. The highest prevalence of HPV genotypes, accounting for 31%, was attributed to the other high-risk genotypes, including 31, 33, 35, 39, 51, 52, 56, 58, 59, 66, and 68, followed by high-risk genotype 16, which counted in 11.60% of cases. Individuals who tested positive for HPV 16 were at a high risk of ASC-H, HSIL, and LSIL. Those testing positive for HPV 18–45 exhibited an elevated risk of LSIL, and those with positive results for other high-risk HPV genotypes were at an increased risk of ASCUS and LSIL, suggesting a low oncogenic potential. The results suggest that the percentage of association between samples with abnormal cervical presentation and negative high-risk HPV diagnosis is noticeably increasing. This underscores the need for effective screening programs and an understanding of the impact of specific HPV genotypes on cervical abnormalities.

## 1. Introduction

Human papillomavirus (HPV) is the most common sexually transmitted infection (STI) prevalent among sexually active individuals worldwide. HPV is an unenveloped, small, double-stranded DNA virus that belongs to the *Papillomaviridae* family [1]. The virus is capable of infecting basal epithelial cells that line the inner surfaces of the cervix, oropharynx, anus, penis, vagina, and vulva [2]. Despite its association with cancer development, HPV infections primarily lead to clinical lesions in the form of warts rather than malignancies.

Research indicates that HPV is attributed to over 90% of anal and cervical cancers, around 70% of vaginal and vulvar cancers, and 60% of penile cancers [3,4,5]. Moreover, recent studies have found that about 60% to 70% of oropharyngeal cancers are linked to HPV infection, often in conjunction with tobacco and alcohol consumption [6]. More than 200 genotypes of HPV have been identified and classified into three genera: Alpha papillomavirus, Beta papillomavirus, and Gamma papillomavirus [7]. Among these, forty genotypes are recognized to be transmitted through sexual contact. HPV is further identified into high-risk (HR) and low-risk (LR) genotypes. The LR genotypes (6, 11, 42, 43, and 44) predominantly target cutaneous sites and can lead to skin warts and condyloma acuminata. The HR genotypes (16, 18, 31, 33, 34, 35, 39, 45, 51, 52, 56, 58, 59, 66, 68, and 70) primarily infect mucosal sites and are associated with malignancies such as vaginal, vulvar, cervical, penile, anal, and oropharyngeal cancers [8].

The global burden of associated HPV infections exhibits significant variation due to diverse factors encompassing geography, socioeconomic conditions, cultural practices, and genetic influences. Moreover, individual intrinsic characteristics, including age, gender, anatomical location, and health status, play a pivotal role in the pathogenesis of HPV-related conditions [9]. Among the most commonly linked cancers to HPV infection are cervical cancers, encompassing both squamous and glandular types in women and oropharyngeal cancers in men [10]. In several countries, HPV-associated oropharyngeal cancers are witnessing an upward trend, particularly among males. In fact, in the United States, HPV-related oropharyngeal squamous cancers have even surpassed cervical cancer in terms of prevalence [11]. A study in the USA looking at the trend of HPV-related oropharyngeal squamous cell carcinoma (OPSCC) found that there were 229,264 adult male and 55,108 adult female cases diagnosed and reported between 2001 and 2018 [12]. 

Globally, cervical cancer ranks fourth among cancers affecting women [9]. The primary risk factor for the development of cervical pre-malignant and malignant lesions is HPV infection. Several secondary factors, including early sexual activity, disadvantaged socioeconomic conditions, smoking, long-term use of birth control pills, and dietary habits, are believed to influence the persistence and progression of HPV infections toward cervical cancer [13]. In Saudi Arabia, cervical cancer is reported to occur less frequently compared to other malignancies affecting women, ranking as the eighth most prevalent cancer among females aged 15 to 44 years. In 2023, the Saudi Public Health Authority (SPHA) released the Saudi Clinical Preventive Guideline. These guidelines included measures for HPV vaccination and screening. The SPHA recommends a two-dose vaccine for girls aged 6–17 against HPV (the Saudi national immunization schedule recommends at ages 11 and 12). Additionally, for women aged between 15 and 26, a catch-up immunization is recommended with three doses (0, 1–2, and 6 months from the first dose) [14]. Furthermore, the SPHA recommends screening for cervical cancer in women after starting sexual activity between the ages of 21 and 65 years with cytology (Pap smear) every three years or, for women aged 30 to 65 years who want to lengthen the screening interval, screening with a combination of cytology and human papillomavirus (HPV) testing every five years [14]. However, it must be mentioned that despite these recommendations, Saudi Arabia lacks a nationwide screening program, leaving about 10.7 million women aged 15 and above with no regular screening and, as a result, at an elevated risk of developing cervical cancer [15]. Testing depends on patients’ preferences or the recommendations of the healthcare provider. Also, cervical cancer screening is recommended after marriage when women become sexually active. This may contribute to a lower incidence of cervical cancer compared to the worldwide incidence. 

Previous studies indicate that the predominant genotypes linked to cervical cancer in the country are HPV 16, HPV 18, and HPV 45, accounting for around 70% of all cervical cancer cases [16]. The prevention of cervical cancer is attainable through comprehensive screening and vaccination programs. In developed nations, increased awareness of HPV screening has led to a decline in cervical cancer incidence [17,18]. However, the extent to which women in Saudi Arabia undergo annual Pap tests remains uncertain. Unfortunately, late-stage diagnosis is still prevalent, resulting in reduced survival rates. 

The present study aims to assess the prevalence of HPV infection and cervical abnormalities among women attending King Fahad Medical City (KFMC) in Riyadh, Saudi Arabia. Furthermore, the study aims to evaluate the existing cervical and HPV screening program and to compare the diagnostic efficiency of HPV genotyping against cytological triage. The overarching goal is identifying genotype-specific risks associated with the progression to abnormal cervical squamous cells.

## 2. Materials and Methods

### 2.1. Study Cohort

This retrospective case-series observational study involved patients who visited King Fahad Medical City (KFMC) in Riyadh, Saudi Arabia, from January 2021 to December 2022. All methods were performed in accordance with the Declaration of Helsinki and the relevant guidelines and regulations.

The study included women with a history of sexual activity who voluntarily attended a gynecological clinic at KFMC for purposes of HPV screening or routine or requested gynecological examinations. For patients with negative cervical screening results, it was recommended to undergo re-screening within a span of 3 to 5 years or whenever symptoms were present. The inclusion criteria comprised two facets: (1) patients with abnormal cervical screening results in accordance with the latest version of the American College of Obstetricians and Gynecologists (ACOG) guidelines for cervical cancer screening and prevention practice and (2) patients who underwent screening for HPV infection. The exclusion criteria encompassed three conditions: (1) pregnancy, (2) negative cervical screening outcomes, and (3) incomplete or insufficient data.

### 2.2. Data Collection

Patients’ age, nationality, and Pap smear findings, including the pathological classification of cervical lesions detected during screening, along with the outcomes of HPV tests, were extracted from the clinical records. This information was sourced from the pathology and clinical laboratory medicine administration database at KFMC. All data related to patients’ personal information, previous interventions for HPV infection such as surgical or destructive treatments of the cervix, as well as any co-morbidities adhered strictly to the stipulated data protection guidelines.

### 2.3. Ethical Consideration

This study was approved by the Institutional Review Board of KFMC, Riyadh, Saudi Arabia (Ref. No. 23-053E). Patients’ details were kept anonymous and confidential.

### 2.4. Procedures 

Liquid-based cytology (LBC): Cytologic preparations were made using the BD SurePath™ liquid-based Pap test. Cervical specimens were taken from the exocervix and the cervical canal and collected using a broom-like device with a detachable head. The detachable head was placed in a collection vial containing 10 mL of ethanol-based preservative fluid. At the laboratory, received vials were vortexed at 3000 rpm for 30 s, followed by centrifugation to separate out the cells from the suspension. Isolated cells were resuspended in a 4 mL sucrose density gradient followed by slide transfer using gravity for adherence. The BD PrepStain system performed automated slide preparation and staining steps for the thin layer preparation of cytologic material.

Cytotechnologists read the slides, with final verification of abnormal results performed by a pathologist. The results were reported according to the 2014 Bethesda System for Reporting Cervical Cytology: squamous cell abnormalities were classified as atypical squamous cells of undetermined significance (ASCUS), atypical squamous cells cannot exclude high-grade squamous intraepithelial lesion (ASC-H), low-grade squamous intraepithelial lesion (LSIL), high-grade squamous intraepithelial lesion (HSIL), or squamous cell carcinoma (SCC). In the Bethesda System, koilocytes (koilocytosis) and cervical intraepithelial neoplasia (CIN 1) were considered LSILs. HSILs include CIN 2 and CIN 3. Negative or unsatisfactory results were included under unsatisfactory for evaluation. Smears with ASCUS, ASC-H, LSIL, and HSIL were sent for HPV screening. Cases in which HPV testing was not performed were excluded from the study. KFMC follows the ASCCP Risk-Based Management Consensus Guidelines for Abnormal Cervical Cancer Screening Tests and Cancer Precursors. 

Sample condition and storage: Cytological evaluation and HPV screening were performed using the same liquid-based cytology sample. 

LBC collection media. Cervical cell samples were stored in a liquid transport medium. Specimens were stored at 5–30 °C during transportation and were kept at room temperature for up to two weeks. Approximately 1–2 mL of specimen was used for each test. 

HPV Screening: Xpert HPV assay was used to qualitatively detect the E6/E7 region of the viral DNA genome from high-risk HPV in patient specimens. The test carried out multiplexed amplification of target DNA using real-time PCR of 14 high-risk HPV types in a single analysis. Xpert HPV specifically identified types of HPV 16 and HPV 18/45 in two distinct detection channels and reported 11 other high-risk types (31, 33, 35, 39, 51, 52, 56, 58, 59, 66, and 68) in a pooled result. The assay was performed on Cepheid GeneXpert Instrument Systems, which automates and integrates sample processing, cell lysis, purification, nucleic acid amplification, and detection of the target sequences in clinical samples. 

The results were interpreted as follows: HPV-16-positive, HPV-18- or 45-positive, HPV others high-risk positive (including: 31, 33, 35, 39, 51, 52, 56, 58, 59, 66, and 68), HPV 18–45 and others high-risk positive, HPV high-risk negative, and HPV high-risk equivocal. 

### 2.5. Statistical Analysis

All data were statistically analyzed using IBM SPSS statistics. Contingency tables were used for the main variables, e.g., HPV infection, age, Pap test results, and cytological diagnosis for each patient, with the values considered separately and in pairs. Pearson’s chi-square test evaluated the correlation between the variables. Frequencies and percentages were calculated for categorical variables, while means and standard deviations were measured for continuous variables. Regressions and linear regressions were performed for age mean vs. HPV PCR (dependent variable: age; reference group: HPV high-risk negative). Categorical data were presented as frequencies and percentages and analyzed using the chi-square test or Fisher’s exact test, as appropriate. The continuous data were tested for normal distribution using the Kolmogorov–Smirnov test and analyzed using the Student’s *t*-test or ANOVA. *p*-values < 0.05 were considered statistically significant. Coefficients for predictor variables in the linear regression analysis were determined, with their uncertainty captured through the 95% confidence intervals computation. For the multinomial logistic regression, relative risk ratios were established alongside their 95% confidence intervals, delineating the expected range of the true values with a 95% confidence level.

## 3. Results

**Figure 1:**
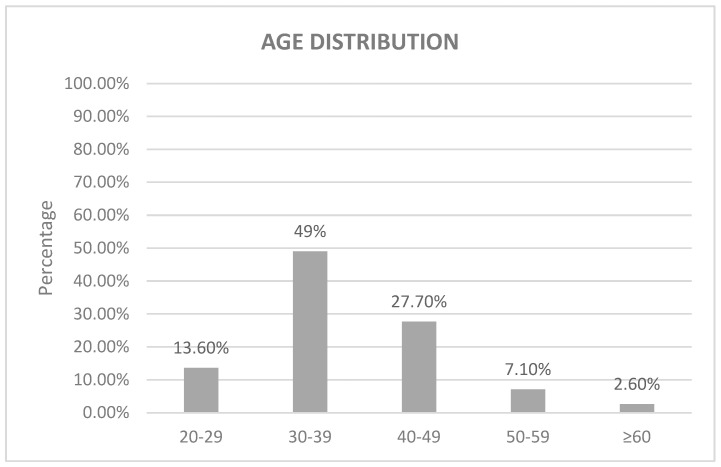
The graph shows the age distribution of patients diagnosed with abnormal cervical smears and screened for HPV infection at KFMC from 2021 to 2022. The majority of patients were aged 30–39 (49%), while those 60 years and above were the minority with only 2.6%.

A total of 155 samples were subjected to statistical analysis obtained from patients who had received diagnoses of abnormal cervical smears and had undergone screening for HPV infection. Of these samples, 88.39% (n = 137) were of Saudi Arabian nationality, whereas the remaining 11.61% comprised individuals from Egyptian, Indonesian, Kuwaiti, Filipino, Syrian, and Yemeni nationalities (n = 2, 1, 1, 11, 1, and 2, respectively). Regarding age distribution, the preponderance of patients, accounting for 49% (n = 76), fell within the age bracket of 30–39. Subsequently, 27.70% (n = 43) were in the age range of 40–49, followed by 13.60% (n = 21) in the 20–29 age category. The subsequent groups were represented by 7.10% (n = 11) for the 50–59 age range and 2.60% (n = 4) for individuals aged 60 and above (Figure 1). 

**Figure 2:**
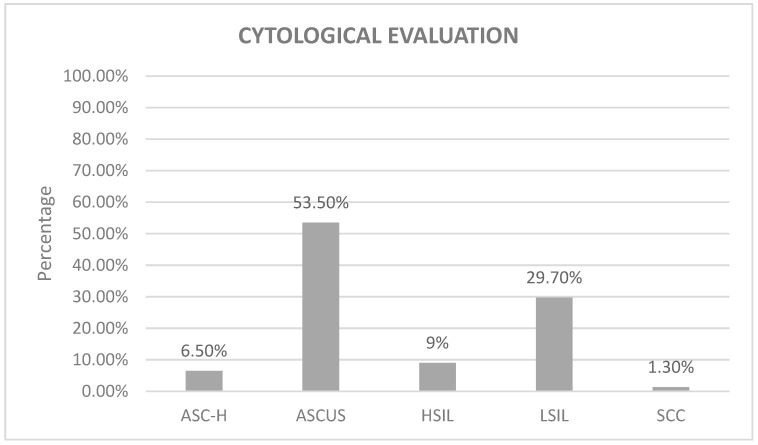
The graph shows the cytological evaluation of patients diagnosed with abnormal cervical smears and screened for HPV infection at KFMC from 2021 to 2022. The most common result was ASCUS with 53.5%, followed by LSIL at 29.7%, then HSIL at 9%, followed by ASC-H at 6.5%, while 1.3% of the samples showed SCC.

Upon scrutinizing the cervical dysplasia within the samples, the cytological assessment revealed ASCUS to be the prevailing interpretation, accounting for 53.50% (n = 83). Successively, LSILs manifested as the subsequent most frequent condition at 29.70% (n = 46), followed by HSILs with a prevalence of 9% (n = 14). ASC-H was observed in 6.50% (n = 10) of cases, and SCCs constituted a minority presence at 1.3% (n = 2) (Figure 2).

**Figure 3:**
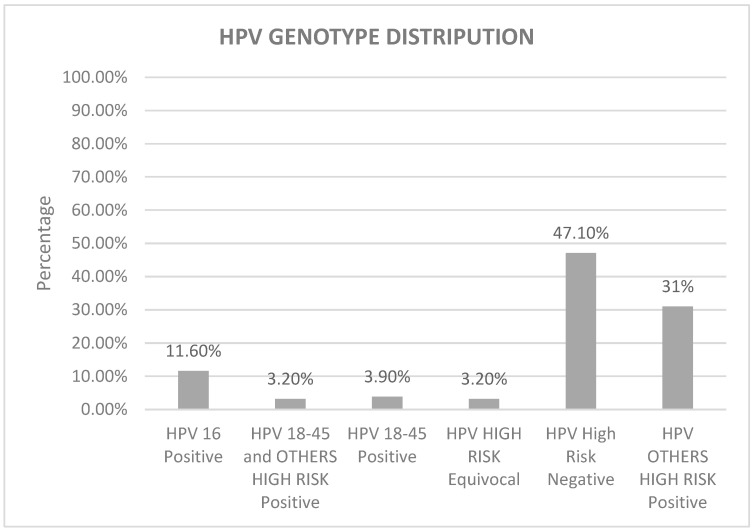
HPV DNA was tested using Cepheid Xpert assay. The majority of samples were negative for high-risk HPV (47.1%). A total of 31% were positive for types other than 16, 18, and 45. HPV-16-positive was reported in 11.60% of cases, while HPV 18–45 showed 3.2% and HPV-18-45-positive with other types was 3.9%. Other high-risk includes 31, 33, 35, 39, 51, 52, 56, 58, 59, 66, and 68.

The HPV test yielded a positive result in 52.9% (n = 82) of the samples and a negative result in 47.1% (n = 73). The predominant HPV types, as indicated by the Cepheid Xpert HPV test, were HPV other types (including 31, 33, 35, 39, 51, 52, 56, 58, 59, 66, and 68), either alone at 31% (n = 48), or in combination with HPV 18–45 at 3.2% (n = 5). Notably, 11.6% (n = 18) of the samples exhibited positive findings for HPV 16, while 3.9% (n = 6) tested positive for HPV type 18–45. Equivocal results for HPV high-risk were observed in 3.2% (n = 5) of the samples (Figure 3).

The application of a one-way ANOVA revealed a noteworthy and statistically significant correlation between age and HPV PCR (*p*-value < 0.05) (index 1). Subsequently, a linear regression analysis conducted on the mean age in relation to HPV PCR highlighted a connection wherein HPV 16 positivity was notably linked with a comparatively younger age group, as opposed to HPV HR negativity (*p*-value = 0.012, coefficient = −5.99). Furthermore, it was observed that HPV other HR positivity was discernibly associated with age groups up to 49 years (*p*-value = 0.029, coefficient = -3.67) (Table 1).

Upon investigating the relationship between age and Pap smear results, the one-way ANOVA yielded a notably significant statistical outcome (*p*-value < 0.01). The predominant diagnostic category among the samples was ASCUS, encompassing 53.5%, followed by LSIL at 29.7%; in contrast, the incidence of SCC was minimal at 1.3%. Furthermore, it was observed that patients afflicted with SCC tended to belong to an older age group in comparison to those diagnosed with ASCUS (Table 2). 

For the assessment of disease risk, a multinomial regression approach was employed, revealing statistically significant associations. Notably, individuals testing positive for HPV 16 exhibited an elevated risk for ASC-H (RRR: 21.8; *p*-value = 0.001; 95% CI: 3.266 to 145.2), HSILs (RRR: 19.6; *p*-value = 0.000; 95% CI: 3.71 to 103.4), and LSILs (RRR: 5.4; *p*-value = 0.031; 95% CI: 1.163 to 25.49). Moreover, other HPV HR types also exhibited statistically significant associations with an augmented risk of LSILs (RRR: 2.6; *p*-value = 0.022; 95% CI: 1.145 to 5.96) (Table 3).

## 4. Discussion

In Saudi Arabia, cervical cancer stands as the eighth most frequent cancer among women aged between 15 and 44 years. Current estimation in Saudi Arabia reveals that every year, around 358 women are diagnosed with cervical cancer and 179 succumb to the disease [16,19,20]. Saudi Arabia has a population of around 10.3 million women aged 15 years and older who are at risk of developing cervical cancer [21]. A retrospective case-control study performed between 2008 and 2011 at King Abdulaziz Medical City, Riyadh, Kingdom of Saudi Arabia, reported the incidence of abnormal epithelial cell abnormalities (ECA) in Pap smears to be 4.3% (841/19,650), 91% of the patients had squamous cell abnormalities, while 9% had glandular cell abnormalities [22]. Additionally, a cross-sectional study performed at King Faisal Specialist Hospital and Research Center, Riyadh, collected a total of 3346 patients’ data between 2002 and 2017 and found that 2.2% had abnormal Pap smear results. Atypical squamous cells of unknown significance were the most frequent abnormality (2%), followed by abnormal glandular cells (0.8%), and 6.5% of the abnormal Pap smear were associated with positive high-risk HPV [23].

In contrast to Western nations, Saudi Arabia’s approach to HPV preventative vaccination remains in a nascent stage. The introduction of the HPV vaccine to young schoolgirls (aged 9–14) was a recent development by the Saudi Ministry of Health (MOH). However, its adoption has encountered resistance due to sociocultural factors [15]. Furthermore, the screening of cervical cancer in Saudi Arabia heavily relies on Papanicolaou (Pap) smears, which are conducted in both private and governmental medical institutions. While Pap smears can effectively serve as screening tools for detecting cervical pre-malignant and malignant conditions, it is essential to note that they are designed primarily for screening purposes and not for definitive diagnosis of HPV infection. The updated WHO cervical cancer prevention guideline recommends that, for the general population of women, HPV DNA detection serves as the primary screening method from the age of 30, with regular testing scheduled every 5–10 years. Conversely, for women who are HIV-positive, the guideline advises HPV DNA detection to commence at the age of 25 years, with regular screenings performed every 3 to 5 years [24].

Australia was one of the first countries to completely transition its national cervical screening program. In December 2017, the entire country switched from cytology (cervical or Pap smear) testing to primary HPV testing every five years with HPV 16/18 genotyping and liquid-based cytology [25]. As of 2020, numerous European countries have already reported the implementation of HPV DNA-based cervical cancer screening. This includes countries such as the Netherlands (fully implemented), Turkey (fully implemented), the United Kingdom (fully implemented in England, Wales, and Scotland and partially in Northern Ireland), Italy (regional implementation), Sweden (regional implementation), Finland (regional implementation), and Spain (regional implementation). Furthermore, several other countries are currently in the process of implementing such programs, including the United States of America, Norway, Denmark, Belgium, Germany, and Malta [26]. Consequently, more countries will soon face the decision of whether to adopt HPV DNA-based cervical cancer screening and determine the most beneficial strategies for their implementation.

The absence of national HPV-based screening programs can lead to a reduction in awareness concerning HPV-related clinical complications not only in the general population but also among HPV-seropositive individuals. Additionally, there is a lack of accurate data regarding the proportion of women who undergo Pap smear screenings annually in Saudi Arabia. Moreover, the number of publications focusing on squamous cell abnormalities and HPV infection in Saudi Arabia remains limited.

This study is designed with the objective of assessing the prevalence of HPV infection and cervical abnormalities. The study also seeks to analyze and compare the diagnostic effectiveness of HPV genotyping against cytological screening within KFMC, a prominent MOH hospital located in Riyadh. This comparison will aid in identifying genotype-specific risks associated with disease progression to cervical squamous cell abnormalities. The study holds the potential to provide insights into disease prognosis and its patterns of dissemination. Additionally, it will contribute to updating the epidemiological data on cervical cancer and HPV infection rates in Saudi Arabia.

In this study, the predominant composition of the samples stemmed from Saudi patients, a characteristic attributed to the inherent nature of the hospital’s referral system. The concentration of cases within the age range of 30–39 can be attributed to the prevailing patterns of sexual behavior in the country. It is worth noting that among the various HPV types, the age group most commonly affected is 30–39, except for HPV 18–45, where the age groups of 20–29 and 40–49 emerge as the most prevalent.

Outcomes presented in Table 1 indicate an association between cervical abnormalities and HPV infection in 52.9% (n = 82) of the samples, with 47.1% (n = 73) testing negative for high-risk HPV. These findings align with an expanding body of evidence suggesting that, in many cases, a lack of complete concurrence between cytological and HPV DNA-based test outcomes often exists. It has been postulated that a positive result from a high-risk HPV DNA test does not consistently correlate with precancerous or cancerous Pap cytology results. For instance, Richardson et al. recently highlighted a decline in the diagnostic accuracy of Pap cytology when evaluating patients’ cervical HPV statuses [27]. This phenomenon might be due to heightened awareness of potential abnormalities, resulting in a higher occurrence of false-positive results. The researchers compared original cytotechnologists’ Pap assessments, wherein HPV status was concealed, and Pap assessments, where HPV status was revealed, across three screening populations. In a study conducted by Lee et al., it was discovered that a negative Pap cytology was detected in nearly 40% of HPV-31-positive samples and 71% of HPV-45-positive samples [28]. Conversely, only about 20% of HPV-16-positive samples detected through a single test at the time of entrance exhibited HSIL cytology. These results could be explained as the majority of HPV infection is transient, where dysplasia is not always presented. Also, it could result from sampling or interpretation bias. 

The prevailing type identified in this study falls under the category of “other high-risk,” encompassing genotypes 31, 33, 35, 39, 51, 52, 56, 58, 59, 66, and 68. Subsequently, the high-risk type 16 follows. This contrasts with prior studies that indicated HPV 16, HPV 18, and HPV 45 as the most commonly associated genotypes within the Saudi population [29]. Our finding agrees with the most updated figure of HPV genotype distribution worldwide [30]. This divergence might underscore the necessity for a broader and more comprehensive study to reassess the current screening system. Such a study should encompass a wider variety of genotypes to ascertain which specific type indeed holds the highest prevalence. It is noteworthy that our dataset indicates ASCUS as the most frequent interpretation. This observation corresponds with the employment of Pap smears for screening, in contrast to HPV DNA tests, as observed in other studies [31,32]. Hence, there is a need to assess the effectiveness of incorporating HPV DNA screening. 

Additionally, our data demonstrate that individuals who tested positive for HPV 16 face a heightened risk of ASC-H, HSILs, and LSILs. Meanwhile, those who tested positive for HPV 18–45 exhibit a potentially elevated risk of LSILs, and those who tested positive for other high-risk HPV types are more susceptible to ASCUS and LSILs. These results suggest low oncogenic potential for HPV 18–45 and other high-risk HPV genotypes. Although the sample size was relatively limited, an association was observed between SCC and negative HPV status, which aligns with published studies indicating a concordance between negative HPV results and SCC [33,34]. It is worth noting that those diagnosed with SCC tend to be of an older age compared to patients diagnosed with ASCUS, which might influence the outcomes [35]. 

The study findings emphasize the importance of implementing effective screening programs and understanding the role of specific HPV genotypes in the development of cervical abnormalities. It is essential to acknowledge the limitations inherent in our study. Firstly, the molecular testing for HPV was conducted in a reflexive manner, which could lead to a notably higher count of HPV-positive cases within our cohort. Consequently, there is also an expected significant proportion of cases categorized as reactive ASCUS, where the corresponding molecular testing yielded negative results. Secondly, our molecular testing protocol employing Xpert HPV, which solely detects high-risk HPV in patient samples. Therefore, the negative HPV outcomes within our cohort may potentially relate to other types of moderate/low-risk HPV infections. Thirdly, a notable limitation arises from the relatively modest sample size, which becomes evident through the presence of “0.00” frequencies in frequency tables. This limited sample size might impact the statistical power and precision of the results obtained. 

In conclusion, the present study determined the prevalence of HPV infection and cervical abnormalities among women attending a tertiary care center in Saudi Arabia over a 2-year timeframe. The results suggest that the percentage of association between samples with abnormal cervical presentation and negative high-risk HPV diagnosis is noticeably increasing. This is a critical finding as it could suggest that there is a shift in the associated cervical abnormality and HPV genotype, where other HPV strains, which are not detected by the test, are involved. Our study also highlighted the importance of accurate molecular diagnostic techniques for HPV detection and identification, critical for diagnosing at-risk patients. This underscores the need for effective screening programs and an understanding of the impact of specific HPV genotypes on cervical abnormalities. 

## Figures and Tables

**Table 1:** Age vs. HPV PCR.

HPV PCR N (%)	HPV 16 Positive	HPV 18-45 Positive	HPV 18-45 + Other HR Positive	HPV Other HR Positive	HPV HR Equivocal	HPV HR Negative	Total
**20–29**	4 (2.6%)	2 (1.3%)	1 (0.6%)	9 (5.8%)	2 (1.3%)	5 (3.2%)	23 (14.8%)
**30–39**	10 (6.5%)	1 (0.6%)	2 (1.3%)	23 (15%)	2 (1.3%)	37 (24%)	75 (48.7%)
**40–49**	4 (2.6%)	3 (1.9%)	2 (1.3%)	15 (9.7%)	1 (0.6%)	20 (12.9%)	45 (29%)
**50–59**	0 (0%)	0 (0%)	0 (0%)	1 (0.6%)	0 (0%)	7 (4.5%)	8 (5.1%)
**≥60**	0 (0%)	0 (0%)	0 (0%)	0 (0%)	0 (0%)	4 (2.6%)	4 (2.6%)
**Total**	18 (11.6%)	6 (3.9%)	5 (3.2%)	48 (31%)	5 (3.2%)	73 (47.1%)	155 (100%)

HR: High-risk.

**Table 2:** Age vs. Pap smear results.

GYN Interp N (%)	20–29	30–39	40–49	50–59	≥60	Total
**ASC-H**	2 (1.3%)	5 (3.2%)	2 (1.3%)	0 (0%)	1 (0.6%)	10 (6.5%)
**ASC-US**	12 (7.7%)	40 (26%)	22 (14.2%)	8 (5.2%)	1 (0.6%)	83 (53.5%)
**HSIL**	1 (0.6%)	7 (4.5%)	4 (2.6%)	1 (0.6%)	1 (0.6%)	14 (9%)
**LSIL**	6 (4%)	24 (15.5%)	14 (9%)	2 (1.3%)	0 (0%)	46 (29.7%)
**SCC**	0 (0%)	0 (0%)	1 (0.6%)	0 (0%)	1 (0.6%)	2 (1.3%)
**Total**	21 (13.6%)	76 (49.2%)	43 (27.7%)	11 (7.1%)	4 (2.4%)	155 (100%)

**Table 3:** HPV PCR vs. Pap smear results.

GYN Interp N (%) *p*-Value	ASC-H	ASC-US	HSIL	LSIL	SCC	Total
**HPV 16**	4 (2.6%)	3 (2%)	6 (3.9%)	5 (3.2%)	0 (0%)	18 (11.7%)
**HPV 18-45**	1 (0.6%)	1 (0.6%)	1 (0.6%)	3 (2%)	0 (0%)	6 (3.8%)
**HPV 18-45 + Other HR**	0 (0%)	2 (1.3%)	0 (0%)	3 (2%)	0 (0%)	5 (3.3%)
**HPV Other HR**	1 (0.6%)	25 (16.1%)	2 (1.3%)	20 (12.9%)	0 (0%)	48 (30.9%)
**HPV HR Equivocal**	1 (0.6%)	3 (2%)	0 (0%)	0 (0%)	1 (0.6%)	5 (3.2%)
**HPV HR Negative**	3 (2%)	49 (31.6%)	5 (3.2%)	15 (9.7%)	1 (0.6%)	73 (47.1%)
**Total**	10 (6.4%)	83 (53.6%)	14 (9%)	46 (29.8%)	2 (1.2%)	155 (100%)

HR: High-risk.

## Data Availability

The data that support the findings of this study are available on request from the corresponding author L.F.
